# Liquid base cytology in evaluation of thyroid nodules

**DOI:** 10.1186/s40200-014-0082-5

**Published:** 2014-09-10

**Authors:** Elahe Keyhani, Sasan A Sharghi, Rana Amini, Sina A Sharghi, Masoud Karimlou, Fatemeh A Moghaddam, Bagher Larijani

**Affiliations:** Genetics Research Center-University of Social Welfare and Rehabilitation Sciences, Koudakyar st.-Daneshjoo blv., Tehran, (1985713834) Iran; Endocrinology and Metabolism Research Center, Tehran University of Medical Sciences, Tehran, (1411413137) Iran; Sepid Pathobiology Laboratory, No.831-North Taleghani Blv., Karaj, (3155783618) Iran; Iran University of Medical Sciences-Hemmat Highway, Tehran, (1449614535) Iran; Social Department of Health Research Center, Department of Biostatistics, University of Social Welfare and Rehabilitation Sciences, Tehran, (1985713834) Iran

**Keywords:** Thyroid fine needle aspiration, Conventional smears, Cell block preparation, Liquid base preparation

## Abstract

**Background:**

Palpable thyroid nodules are present in 4-7% of general population and Fine Needle Aspiration (FNA) is now accepted by endocrinologists and thyroid surgeons as a safe, simple and cost effective procedure for evaluating a thyroid nodule. The obtained sample can be spread directly on slides, processed as cell block preparations or prepared as liquid base smears. Liquid base method has been recently accepted due to its shorter preparation time and better preservation of nuclear details.

The aim of this study is to compare the diagnostic results of two commonly used methods: Liquid Base Preparation and Cell Block Preparation in evaluation of thyroid nodules.

**Methods:**

The samples were taken from 100 patients with a solitary nodule or a prominent nodule on a multinodular goiter background (excluding hot nodules). The obtained samples were used to prepare conventional smears (CS), Cell Block Preparations (CBP) and Liquid Base Preparations (LBP). The slides were studied by two pathologists, considering the following parameters: Cellularity, Colloid, Lymphocytes/Plasma cells and Macrophages.

**Results:**

87% of cases revealed informative results in LBP method while in the same group of patients only 69% of samples were informative after processing by CBP method. Sensitivity and specificity of both methods compared with the conventional smears and with each other and it is concluded that LBP is a reliable method for evaluating of a thyroid nodule. Other studies also show the same results.

**Conclusion:**

The liquid base method should be trusted due to its easier procedure, cleaner slide background, its higher specificity and higher diagnostic yields. It can be used instead of CBP and in association with CS to increase the accuracy of evaluation of thyroid nodules.

## Background

Palpable thyroid nodules are common clinical findings which are found in 4-7% of general population, postmoterm studies (autopsies) show thyroid nodules above 1 cm diameter in about 50% of general population and ultrasonography reveal 67% prevalence of nodules of any size [1].Although the clinical criteria especially soft consistency of nodule and its liquid content can be of help to reduce the possibility of malignancy [2], definite diagnosis is based only on microscopic findings. FNA (Fine Needle Aspiration) is accepted as a simple, relatively safe and cost effective procedure which provide material for microscopic evaluation [3]. The obtained material can be processed in three ways: −Constituitional smears (CS): simple, thin smears (fixed or air dried). -Cell block preparations: tissue sections of paraffin embedded concentrated aspirates. –Liquid base preparations (LBP): smears obtained from processed and concentrated samples [4,5]. Since most of the nodules are benign, FNA could decrease the rate of unnecessary surgeries [6] and save them only for suspicious and malignant lesions. The aim of this study is to compare the diagnostic results of two commonly used methods (LBP & CBP) in evaluating of the thyroid nodules.

## Patients and methods

One hundred patients with a palpable thyroid nodule equal or more than 1 cm in diameter or a *prominent nodule* on a multinodular background (excluding hot nodules) were selected. A prominent nodule is refered to a nodule which is suspected for malignancy, clinically or according to sonographic criteria. FNA was performed for all patients, without ultrasound guidance, using 23 guage needle and at least 3 needle passes.

The obtained specimens were used to: 1-Prepare conventional smears (CS), alcohol-fixed for papaniculaou staining and air-dried for geimsa staining. 2-Prepare CBP and LBP slides: The aspirated material was rinsed in a cytofixative solution (Liqui-PREP preservative solution-LGM international Inc.) and divided into two parts:

- Part 1 for cell block preparation:
  - After centrifugation at 2500 rpm (rate per minute), the precipitants were placed on a piece of filter paper and passed the fixation procedure according to Shidman’s standard protocol [7]. The fixed specimens then used for preparing the paraffin blocks, were cut into 4–5 μ thickness and stained by Hematoxillin & Eosin according to standard protocol [8].
- Part 2 for liquid base preparation:
- The samples were stayed at least 1 hour in room temperature with preservative in order to be fixed.
- Equal volume of lytic solution (Liqui-PREP cleaning solution-LGM international Inc.) was added to the sample and after 30 seconds mixing, remained for 30 minutes and then centrifuged 10 minutes with 2500 rpm.
- The supernatant was discarded.
- 50-100λ of cell base (Liqui-PREP cleaning solution-LGM international Inc.), based on the pellet size, was added and mixed. Then thin layer smears were prepared using 100λ of the sample.
  ❖ The remaining solution could be used for further studies such as Immunostains (Immunocytochemistry), if necessary.
- After 1 h in room temperature, the prepared smears were fixed by 95% alcohol for 15 minutes.
- Papaniculaou staining was performed according to standard protocol [9].
- coverslips were attatched.

Two pathologists studied all of the slides, considering the following elements: Cellularity (score 0 to 4), Colloid (score 0 to 4), Lymphocytes/Plasma cells (score 0 to 4) and Macrophages (score 0 to 4). Minimally 5 groups of 10 thyroid native cells were considered as sufficient (informative) and less cellularity as insufficient (non-informative).The microscopic findings of two pathologists revealed a high interobserver agreement. In the few cases of disagreement (2 patients out of 100) the slides were studied jointly and discussed to obtain an agreed same result, considering the diagnostic criteria. The informative results were categorized as Benign, Suspicious and Malignant according to Bethesda system classification [10] (Table 1).

**Table 1:** **Bethesda system for Reporting Thyroid Cytopathology**

1	Non-Diagnostic
2	Benign
3	Suspicious
*Atypia of Undetermined Significance	
(Follicular Lesion of Undetermined Significance)	
*Follicular Neoplasm	
*Suspicious for Malignancy	
4	Malignant

The informed consent forms were obtained from all of the participants for the publication of this report and the related images. This study has been approved by the ethics committee of Tehran University of Medical Sciences.

## Results

**Figure 1:**
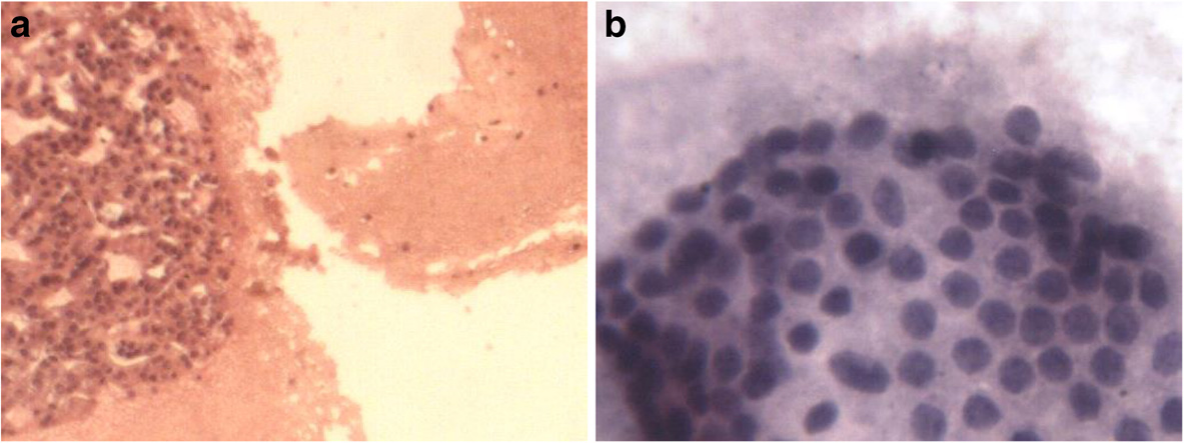
Cell block preparation (a) and Liquid base preparation (b) comparative illustrations-*400 magnification-(E. Keyhani et al.).

87% of cases revealed informative results in LBP method while in the same group of patients only 69% of samples were informative after processing by CBP method (Table 2). In 31% of samples LBP and in 13% CBP were more informative and diagnostic (Figure 1). So in comparison with the conventional method (CS), the sensitivity and specificity of LBP & CBP methods are calculated as followed:

- LBP sensitivity: 95% LBP specificity: 31%
- CBP sensitivity: 96% CBP specificity: 24%

**Table 2:** **The descriptive results**

**Method**	**Informative results**	**Non-informative results**
Liquid Base Preparation	87%	13%
Cell Block Preparation	69%	31%

As mentioned above, both techniques have equal sensitivity, while the specificity of LBP is higher than CBP. When we compare the True Positive, False Positive, False Negative and True Negative parameters of two tests (Table 3), it could be realized that after examining all of the informative cases of CBP by LBP method, 91% of the samples are informative; while subject to informativeness of LBP only 72% of cases were informative by CBP.

**Table 3:** **Comparitive table of LBP & CBP**

	**Informative LBP**	**Non-informative LBP**	**Sum**
Informative CBP	63 (True Positive)	6 (False Positive)	69
Non-informative CBP	24 (False Negative)	7 (True Negative)	31
Sum	87	13	100

According to Table 3, if all of the cases whom are non- informative in CBP method examined by LBP method, 77% will be informative while subject to non-informative cases in LBP method only 46% of cases were informative using CBP (Table 3); so LBP could be considered as a more reliable test than CBP for evaluating of thyroid nodules.

## Discussion

The incidence of thyroid nodules has been raised in recent years, probably due to wider application of thyroid imaging techniques [11]; the increased rate encourages the physicians to apply more reliable procedures to evaluate this lesions.

Our study which is the first study in Iranian population showed that the application of LBPs added to CS, in comparison with CBPs, increased the diagnostic value of FNA technique and therefore is preferred over CBP for the evaluation of thyroid nodules.

In our study, all three techniques (CS, LBP, and CBP) performed for the same group of patients, in contrast to the most other studies which has selected different groups of patients, each group for each technique.

FNA is the first step diagnostic tool in thyroid nodule evaluation [1,6]. It could prevent unnecessary thyroid surgeries [12]. In the recent years LBP cytology is widely used and is replacing the CS [13] especially in gynecological samples [14]. The application of LBP instead of CBP is preferred by some authors; Saleh et al. in 2008 reported a comparative study of 126 cases of thyroid CS & 128 cases of LBP [11].

The authors used a semiquantitative scoring system considering Cellularity (0–4), Colloid (0–4), Macrophages(0–4), Lymphocytes/Plasma cells (0–4) and reported a higher diagnostic rate for LBP (68% for LBP & 24% for CB slides) [15].

In another study on 2523 LBPs and 1767 CSs FNA sensitivity,specificity, positive predictive value (PPV) and negative predictive value (NPV) of both methods were reported nearly equal, in that study the diagnostic value of both techniques were the same, but the rate of indeterminate and ACUS diagnosis was lower in LBP method;meanwhile LBPs revealed more clear nuclear details and cleaner background [13]. The most important advantages of CBPs are described as better tissue architecture preservation, the ability of preparing multiple sections and the capacity to apply Immunohistochemistry (IHC) stains, but the method is time-consuming and more expensive in comparison with LBPs [6,16]. A few years later some studies showed that immunostains can be applied on LBPs and this method is becoming more popular for evaluating non-gynecological samples [15]. Decreased obscuring background elements such as mucus, inflammatory elements and blood makes a cleaner background and eases study of the smears. Meanwhile the residual sample in the fixative solution allows to examine different parts of the specimen and application of ancillary studies such as Immunostaining, flowcytometry and other studies, if necessary [17]. Using LBP also helps to scape from many of the CS artifacts [16]. Perfect interobserver agreement on LBP results between three pathologists (considering the kappa test) in Tettikurt’s study mentioned the reproducibility of LBP test [12]. In another comparative study between CSs and LBPs in 2013, the authors showed that for cases with a “benign reference diagnosis” LBPs performed better than CSs, however for cases with a reference diagnosis of “papillary thyroid carcinoma” CSs were better.

This study suggests that applying both methods in order to increase accuracy [15]. Another report on gynecological samples from France confirms that CSs and LBPs have the same sensitivity and either could be used as screening method according to local economical considerations [18]. Despite the advantages of LBPs such as faster slide screening capability, low costs,easy procedure, presentation of uniform cells [15], high overall accuracy, preserved fine nuclear details, prominent nucleoli, decrease background noises and allowing to examine the whole specimen [17], the procedure is occasionally used lonely [13,19].

In order to increase the quality of the obtained material and decrease noninformative results, method standardization [3] and ultrasound (US) guided FNA could be effective [20]. US can detect 5 mm nodules but micronodules (less than 1 cm diameter) have a low risk of malignancy or little impact on patients survival; only those micronodules which carry suspicious features at US (such as microcalcification) should be studied by cytopathologist [20]. Level of experience of the pathologist is an important factor for interpreting the LBP samples [15]. The morphological details are somewhat different between the two methods, smaller fragments in LBP and cell dyshesion [17] are the main challenges which the pathologist should be familiar with them, and awareness of cytomorphologic changes in LPB method is needed to avoid misinterpretation [15].

## Conclusion

Since the combination of minimally two methods of FNA preparations increase the accuracy, it is suggested to use CS in association with LBP in all aspirated specimens of thyroid FNA. The possibility of applying immunostains and even flowcytometry on LBP smears help to have benefits of all diagnostic tools.

## Abbreviations

FNA: Fine needle aspiration
CS: Conventional smear
CBP: Cell block preparation
LBP: Liquid base preparation
Rpm: Rate per minute
PPV: Positive predictive value
NPV: Negative predictive value
IHC: Immunohistochemistry
